# Pilot Study on Quantitative Cervical Cord and Muscular MRI in Spinal Muscular Atrophy: Promising Biomarkers of Disease Evolution and Treatment?

**DOI:** 10.3389/fneur.2021.613834

**Published:** 2021-03-29

**Authors:** Giovanni Savini, Carlo Asteggiano, Matteo Paoletti, Stefano Parravicini, Elena Pezzotti, Francesca Solazzo, Shaun I. Muzic, Francesco Santini, Xeni Deligianni, Alice Gardani, Giancarlo Germani, Lisa M. Farina, Niels Bergsland, Claudia A. M. Gandini Wheeler-Kingshott, Angela Berardinelli, Stefano Bastianello, Anna Pichiecchio

**Affiliations:** ^1^Advanced Imaging and Radiomics Center, Neuroradiology Department, IRCCS Mondino Foundation, Pavia, Italy; ^2^Department of Brain and Behavioral Sciences, University of Pavia, Pavia, Italy; ^3^Department of Radiology, Division of Radiological Physics, University Hospital Basel, Basel, Switzerland; ^4^Department of Biomedical Engineering, University of Basel, Allschwil, Switzerland; ^5^Child Neuropsychiatry Unit, IRCCS Mondino Foundation, Pavia, Italy; ^6^Buffalo Neuroimaging Analysis Center, Department of Neurology, Jacobs School of Medicine and Biomedical Sciences, University at Buffalo, The State University of New York, Buffalo, NY, United States; ^7^IRCCS, Fondazione Don Carlo Gnocchi ONLUS, Milan, Italy; ^8^NMR Research Unit, Queen Square MS Centre, Department of Neuroinflammation, UCL Queen Square Institute of Neurology, University College London, Russell Square, London, United Kingdom; ^9^Brain Connectivity Research Unit, IRCCS Mondino Foundation, Pavia, Italy

**Keywords:** spinal muscular atrophy, muscle MRI, nusinersen (spinraza), motor neuron degeneration, spinal cord atrophy and degeneration, muscle fat fraction, quantitative magnetic resonance imaging, treatment monitoring

## Abstract

**Introduction:** Nusinersen is a recent promising therapy approved for the treatment of spinal muscular atrophy (SMA), a rare disease characterized by the degeneration of alpha motor neurons (αMN) in the spinal cord (SC) leading to progressive muscle atrophy and dysfunction. Muscle and cervical SC quantitative magnetic resonance imaging (qMRI) has never been used to monitor drug treatment in SMA. The aim of this pilot study is to investigate whether qMRI can provide useful biomarkers for monitoring treatment efficacy in SMA.

**Methods:** Three adult SMA 3a patients under treatment with nusinersen underwent longitudinal clinical and qMRI examinations every 4 months from baseline to 21-month follow-up. The qMRI protocol aimed to quantify thigh muscle fat fraction (FF) and water-T2 (w-T2) and to characterize SC volumes and microstructure. Eleven healthy controls underwent the same SC protocol (single time point). We evaluated clinical and imaging outcomes of SMA patients longitudinally and compared SC data between groups transversally.

**Results:** Patient motor function was stable, with only Patient 2 showing moderate improvements. Average muscle FF was already high at baseline (50%) and progressed over time (57%). w-T2 was also slightly higher than previously published data at baseline and slightly decreased over time. Cross-sectional area of the whole SC, gray matter (GM), and ventral horns (VHs) of Patients 1 and 3 were reduced compared to controls and remained stable over time, while GM and VHs areas of Patient 2 slightly increased. We found altered diffusion and magnetization transfer parameters in SC structures of SMA patients compared to controls, thus suggesting changes in tissue microstructure and myelin content.

**Conclusion:** In this pilot study, we found a progression of FF in thigh muscles of SMA 3a patients during nusinersen therapy and a concurrent slight reduction of w-T2 over time. The SC qMRI analysis confirmed previous imaging and histopathological studies suggesting degeneration of αMN of the VHs, resulting in GM atrophy and demyelination. Our longitudinal data suggest that qMRI could represent a feasible technique for capturing microstructural changes induced by SMA *in vivo* and a candidate methodology for monitoring the effects of treatment, once replicated on a larger cohort.

## Introduction

Spinal muscular atrophy (SMA) refers to a spectrum of genetic neuromuscular disorders characterized by the degeneration of alpha motor neurons (αMNs) in the spinal cord (SC) (1) that leads to progressive muscle atrophy, consequent loss of muscular strength and paralysis. Within this group of disorders, the so-called 5q SMA is a rare condition (estimated incidence: from 1/6,000 to 1/10,000 live births) (2) caused by homozygous loss of function of SMN1 gene (survival motor neuron gene 1—telomeric form) (5q11.2-q11.3), with consequent SMN protein deficiency in lower motor neurons. SMA phenotypes encompass a continuum ranging from extremely severe neonatal onset to mild adult onset (3). Phenotypic variability in SMA is largely determined by the copy number of the SMN2 gene. Each SMN2 copy only codes for 10–15% of functional SMN proteins. In all SMA patients, because of the SMN1 deletion, SMN levels in neurons depend on SMN protein coded by the SMN2 gene, which in turn depends on the copy number of SMN2 gene. Therefore, the SMN2 copy number inversely correlates with disease severity (4, 5).

Once the role of the SMN protein expression from SMN2 was recognized, several strategies were developed to increase SMN protein expression (6). Three approved therapies for the treatment of SMA are currently available: nusinersen, a 2-O-methoxyethyl phosphorothioate-modified antisense oligonucleotide that modifies the splicing of the SMN2 pre-mRNA, increasing the production of full-length SMN protein from the SMN2 gene; onasemnogene abeparvovec (AVXS-101), a gene replacement therapy; and risdiplam, an orally administered small-molecule SMN2 splicing modifier that was recently approved by the Food and Drug Administration for the treatment of SMA in adults and children (2 months of age and older). Several other new therapeutic strategies are also currently under study in addition to the aforementioned molecules (e.g., small-molecule splicing modifiers, anti-myostatin agent, and troponin activator) (7). The current availability of such therapies actually requires the scientific world involved in SMA to rapidly identify reliable measures aimed at quantifying and understanding the response to treatment.

Functional scales such as Revised Upper Limb Module (RULM) (8), Hammersmith Functional Motor Scale Expanded (HFMSE) (9), and 6-Minute Walk Test (6MWT) (10) are commonly used to assess the clinical status and to grade the clinical disability in SMA, classifying patients into four different categories (11, 12). *In vivo*, nerve conduction and in particular compound muscle action potential (C-MAP) have been proposed to quantify the degeneration of αMNs (13). Serum levels of SMN protein have also been shown to correlate both with clinical severity and with C-MAP results. Alves et al. (14) have recently demonstrated that serum creatinine level correlates with both C-MAP results and disease severity, making it a candidate biomarker for SMA progression.

Magnetic resonance imaging (MRI) represents an extremely valuable tool that allows non-invasive assessment of both muscle and motor neurons degeneration. Muscle MRI is increasingly used to evaluate muscle involvement in neuromuscular disorders, to identify specific patterns of disease, to guide biopsy, to monitor the progression of the natural disease course and to assess response to therapy (15, 16). In SMA, muscular involvement has been described as a combination of muscle atrophy and muscle fatty substitution. Durmus et al. (17) reported a specific pattern of muscle involvement in 25 subjects with genetically confirmed SMA 3b, with prominent fat replacement evident in the iliopsoas and gluteus maximus muscles and in the triceps and biceps brachii muscles in the upper limbs. In a recent longitudinal study on two siblings with SMA 3b under treatment with nusinersen, fat replacement was assessed using a semi-quantitative method (18) and no significant changes were demonstrated at 10- and 24-month follow-up, presumably due to the elevated degree of fat replacement already present at baseline (19). Only one recent study explored quantitative muscle MRI measurements in SMA 2 and 3 subjects, showing a significant increase of fat fraction (FF) in SMA subjects compared to healthy controls (HCs) (47.6 vs. 7.6%) by using a four-point Dixon sequence, and investigating also water T2 (w-T2) in muscle (20). Two studies already explored the diffusion properties of muscle tissues in SMA by applying diffusion tensor imaging (DTI) cross-sectionally and longitudinally (19, 20), with promising results. To our knowledge, however, longitudinal quantitative muscle MRI data, in particular FF and w-T2, have never been reported in SMA patients undergoing therapy.

Quantitative MRI (qMRI) of the cervical SC has been widely used to investigate cord pathology in several neurodegenerative disorders such as MS (21, 22) and ALS (23, 24). Through the acquisition of images with high contrast between white matter (WM) and gray matter (GM), SC MRI enables tissue segmentation and measurement of the cross-sectional area (CSA) of the SC, total GM, and ventral horns (VHs), thus making it possible to quantify total SC and GM atrophy (25). Advanced techniques such as magnetization transfer (MT) and diffusion tensor imaging (DTI) of the SC can be used for *in vivo* quantification of possible pathology-related microstructural changes occurring within the SC (26). qMRI of the cervical SC has been recently applied in three recent cross-sectional studies focusing on pediatric and adult SMA patients not under therapy (27–29). El Mendili et al. (27) reported a significant cord atrophy gradient with a more evident reduction of SC CSA between C3 and C6 levels of adult SMA patients compared to HCs; Querin et al. (28) investigated both WM and GM, showing a significant reduction of the CSA of both total SC and GM (but not of WM) at each cervical level (from C2 to C7) in adult untreated SMA patients (SMA 3a, 3b, and 4); finally, in the third study, both pediatric and adult SMA patients (type 2 and 3) showed lower SC CSA values than HCs, though the difference was not statistically significant (29). Two of the studies mentioned above also explored the diffusion properties of SC in SMA: Stam et al. (29) reported significantly higher axial diffusivity (AD) in the GM of SMA patients than HCs, while Querin et al. (28) found preserved diffusion properties of SC WM. However, to date and to the best of our knowledge, there are no imaging studies that longitudinally describe the natural evolution of SC features in SMA patients or the effects of treatment on SC properties measured by qMRI over time.

The aim of this pilot study is to apply muscle and spinal qMRI in a small cohort of SMA 3a patients currently being treated with nusinersen at our Institution, in order to establish whether quantitative muscle and cervical SC MRI parameters can provide biomarkers to monitor the course of the disease, understand possible mechanisms of progression or recovery and assess the effectiveness of treatment.

## Methods

### Subjects

In this study we enrolled three female patients affected by genetically diagnosed Type 3a SMA [age at the time of baseline examination—time point 1 (TP1) = 23.0 ± 3.5 years]. Our center provides nusinersen for patients with SMA using intrathecal injections via lumbar puncture, and the dosing schedule consists of four loading doses of 12 mg in the first 2 months of treatment, followed by maintenance doses every 4 months (30). All three patients started treatment at the same time, and the duration of treatment at the time of the last observation time point reported here (TP6) was 21 months. Patients underwent clinical examination at the first loading dose of nusinersen (TP1) and at the time of each maintenance dose. Muscle MRI started to be performed from TP1 as well and was repeated after 9 months (TP3) and 21 months (TP6) from the beginning of therapy. SC MRI examination started at TP3 (9 months after the beginning of treatment, age at TP3 = 24.0 ± 3.5 years) for a total of four time points, each at 4 months from the previous one (TP3 to TP6). Eleven female healthy volunteers, age-matched to that of the SMA patients at TP3 (age 24.5 ± 2.0 years), were also enrolled as HCs for SC MRI only, undergoing a single MRI examination. The study was approved by the local Ethics Committee and written informed consent was given by all participants.

### Clinical Evaluation

At each time point, a neurological examination was performed, and the motor function was assessed through the HFMSE and the RULM tests. The 6MWT was also performed in the only ambulatory patient (Patient 1).

### MRI Acquisition

MR images were acquired with a Siemens 3T MAGNETOM Skyra scanner using an 18-channel surface coil for the thigh muscle MRI, while for MRI of the SC, a 20-channel head/neck coil was combined with the 12-channel spine array integrated into the patient table.

#### MRI of the Thigh Muscles

The muscle MRI protocol was centered on the thighs, with simultaneous acquisition of both sides (total scanning time of approximately 15 min). The MRI protocol included a 3D six-point multi-echo gradient echo (GRE) sequence with interleaved echo sampling (matrix size = 432 × 396 × 52, TR = 35 ms, 6 echo times, TE = 1.7–9.2 ms, ΔTE = 1.5 ms, resolution = 1.0 × 1.0 × 5.0 mm^3^) and a turbo spin echo T2 multi-echo sequence (TE = 10.9 ms, TR = 4100.0 ms, resolution = 1.2 × 1.2 × 10.0 mm^3^, 17 echo times).

#### MRI of the Cervical SC

The SC MRI protocol adopted for this study is derived from the consensus acquisition protocol produced by a consortium of SC researchers [(31); Cohen-Adad et al., Submitted, https://spine-generic.readthedocs.io/en/latest/]. The whole protocol included T1-weighted (sagittal 3D MPRAGE, field of view = 320 × 260 × 192 mm^3^, resolution = 1.0 × 1.0 × 1.0 mm^3^, TI/TR/TE = 1,000/2,000/3.72 ms) and T2-weighted 3D morphological sequences (sagittal 3D SPACE, field of view = 256 × 256 × 52 mm^3^, resolution = 0.8 × 0.8 × 0.8 mm^3^, TR/TE = 1,500/120 ms), with the T1w volume providing full coverage of the brain and the T2w volume centered on the cervical SC at the level of the C3–C4 vertebral disc. These were followed by a DWI acquisition (axial 2D SE-EPI, field of view = 86 × 33 × 75 mm^3^, resolution = 0.9 × 0.9 × 5.0 mm^3^, TR/TE = 620/60 ms, b = 800 s/mm^2^, 30 directions) using the Siemens ZOOMit technology for reduced FOV (32) and cardiac gating a set of three 3D gradient echo sequences for MT imaging [axial, field of view = 230 × 230 × 110 mm^3^, resolution = 0.9 × 0.9 × 5.0 mm^3^, TR/TE = 35(15)/3.13 ms], and a 2D T2^*^-weighted (axial 2D MEDIC, field of view = 224 × 224 × 75 mm^3^, resolution = 0.5 × 0.5 × 5.0 mm^3^, TR/TE = 600/14 ms) scan, all centered at the level of C3–C4 vertebral disc. The total acquisition time for the cervical SC MRI was approximately 30 min, depending on heart rate.

### Analysis of MR Images

#### MRI of the Thigh Muscle

A total of 12 muscle regions of interest (ROIs) were manually drawn by a single experienced operator using ITK-snap v3.0 (33). These ROIs were drawn at the medial level of the thigh (equidistant from the femur head and the tip of the patella), on two central slices of the first echo of the turbo spin echo sequence. ROIs were subsequently registered to the T2 GRE space. Given the considerable fat replacement of the subjects, a unique global ROI including all muscles and fascia was also drawn independently from the previous ROIs for separate evaluation (Global ROI). **Figure 2** reports muscle segmentation of an exemplificative subject (Patient 2 at TP3). The segmented muscles are reported in the figure caption.

The Fatty Riot algorithm was used offline for the calculation of fat/water images from the GRE multi-echo acquisition (34, 35), and then fat fraction maps were obtained.

The images acquired with the multi-echo spin-echo sequence were processed with an extended phase graph (EPG) method for multi-compartment T2 fitting with slice profile correction: this produced a quantitative w-T2 map of thigh muscles (36–38).

Average values of FF and w-T2 were calculated for the global ROI and for each thigh muscle (see **Figure 2**) and, at a later stage, also for each thigh compartment (anterior, medial, and posterior) (39) at TP1, TP3, and at the end of the follow-up (TP6). Given the very small number of subjects, data were qualitatively compared to assess average FF and w-T2 at the different considered time points.

#### MRI of the Cervical SC

All MR images of the cervical SC were processed using the Spinal Cord Toolbox (40) and custom scripts in Matlab (The MathWorks, Inc., Natick, MA, USA). Results were visually inspected by a physicist and a trained neuroradiologist, blinded to clinical data, who evaluated the overall image quality and the presence of artifacts; they also manually corrected segmentation masks and vertebral labeling where an unsatisfactory performance or even a failure of the automatic algorithm made it necessary.

T2w images were used to compute the CSA of the SC from C2 to D3. The SC was segmented using a method based on neural networks (41); it was then manually labeled at the different vertebral levels and registered to the PAM50 template (42).

T2^*^w images were used to compute the CSA of the SC GM and of the VHs from C3 to C4. GM was segmented using a neural network method (43); the template of the SC was registered to T2^*^w images to obtain vertebral labeling and masks of the VH that were intersected with the GM mask. In order to assess possible asymmetries, GM and VH binary masks were also split into left and right side masks using the corresponding atlas labels that were intersected with the respective masks of interest.

T1w images served as anatomical reference and for diagnostic purposes; they were also used to improve the results of template registration with DWI and MT images.

DWI images were corrected for motion and the SC was segmented (41). They were registered to the SC template and the DTI model was fitted to the diffusion data (44). Warped atlas probabilistic labels were used to extract weighted average values of diffusion tensor metrics (fractional anisotropy, FA; mean diffusivity, MD; radial diffusivity, RD; axial diffusivity, AD) for tissues and structures of interest.

The MT set of images was processed to compute magnetization transfer saturation (MTsat) maps to evaluate the SC myelin content (45). Processing included image co-registration, cord segmentation, and image registration to the cord template. Warped atlas probabilistic labels were used to extract weighted average MTsat values of specific SC regions.

SC CSA values were averaged along the whole considered portion of the SC (from C2 to D3), along cervical and dorsal vertebrae separately, and also per vertebral level. Global DTI and MT average values were obtained from ROIs covering C3 and C4 vertebral levels. The ROIs considered for DTI and MT analysis corresponded to cord GM and its subregions [VHs, dorsal horns (DHs), and GM intermediate zone (IZ)] and cord global WM together with three WM bundles: the ventral corticospinal tract (vCST), the lateral corticospinal tract (lCST), and the fasciculus gracilis (FG). The sample standard deviation was calculated for each average value. The resulting CSA, diffusion, and MT values were compared between HC and SMA groups at the first SC MRI time point (TP3) and also longitudinally between successive time points for SMA patients. Given the small number of SMA patients involved in this study, only qualitative comparisons were performed of each patient quantitative metric against the distribution of values obtained in the HC group.

## Results

### Clinical Evaluation

Clinical features of the cohort are summarized in Table 1. All patients showed the typical clinical picture of SMA (hypotonia, weakness, areflexia, fasciculations, ligamentous laxity in hands) and a slowly progressive clinical course of the disease. Notably, Patient 2 had developed a striking asymmetry in strength of upper limbs since the age of 17. Before starting the treatment, none of the patients needed mechanical ventilation, and only Patient 3 developed a moderate scoliosis not requiring spine surgery; such features have not changed over the time of observation.

**Table 1:** Main clinical features of the enrolled patients.

	**Patient 1**	**Patient 2**	**Patient 3**
Age at BL (years)	27	21	21
Age at onset (months)	24–36	12–24	31
Family history	-	-	One maternal uncle affected by genetically proven 5q SMA
Symptoms at onset	Walking impairment, frequent falls, problems in rising from the floor	Asked to be carried, used her upper limbs to better move lower limbs	Waddling gait
Clinical peculiarities	-	Strength asymmetry in upper limbs (left < right)	-
Age at diagnosis (years)	6 (molecular diagnosis)	3	3
SMN2 copies (*n*)	4	3	4
Age at loss of ambulation (years)	- (still ambulatory)	16	19^*^
Respiratory problems/scoliosis	No/No	No/No	No/Yes (no surgery)
Treatment duration at TP6 (months)	21	21	21

*BL, baseline*.

* *Patient 3 started using a wheelchair for outdoor at 4; she was still able to move a few steps with support until the age of 11*.

**Figure 1:**
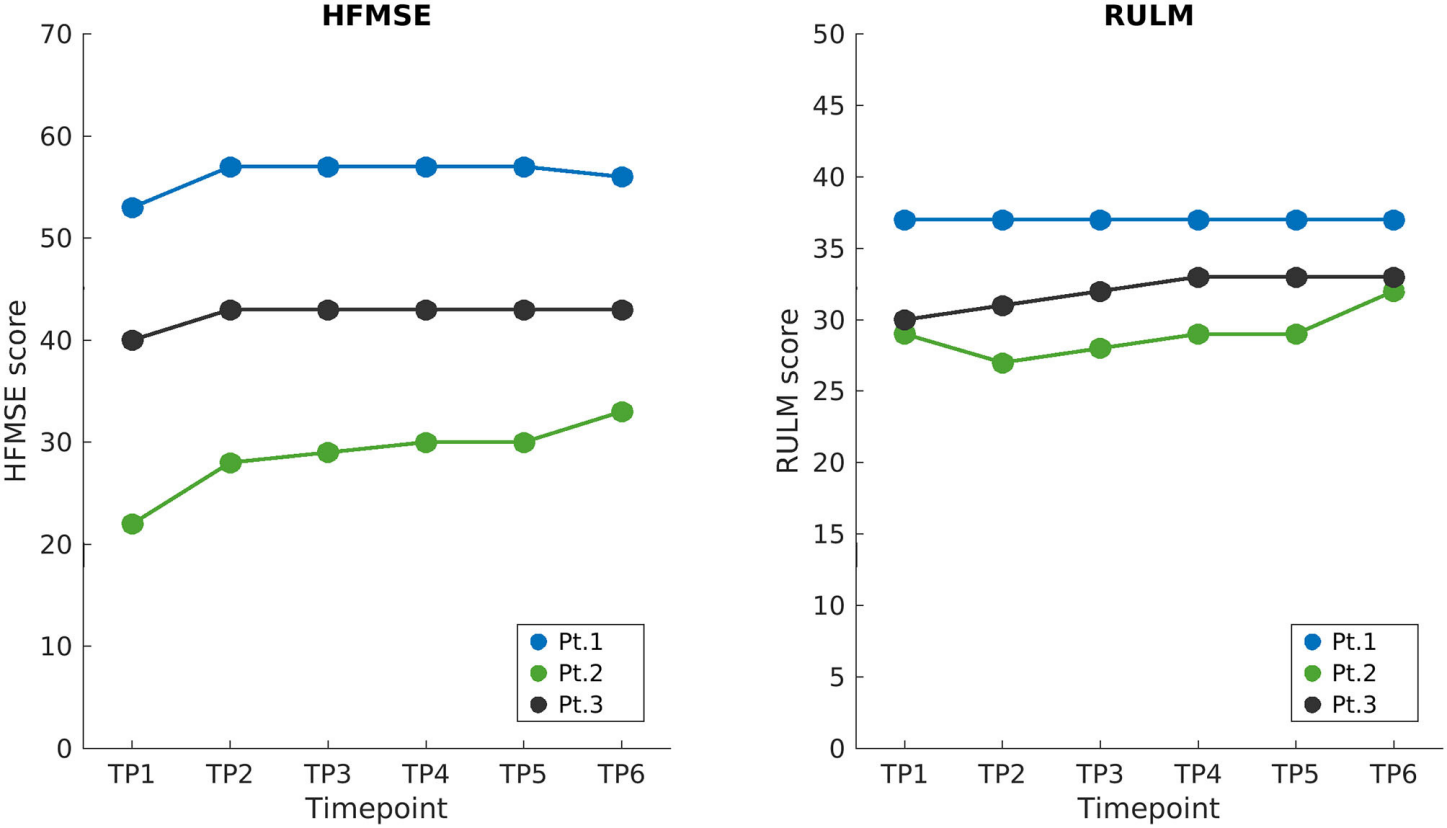
Longitudinal clinical scores. HFMSE and RULM scores of SMA patients are shown on the left and right, respectively, starting from the baseline when treatment began (TP1), up to the end of the follow-up 21 months later (TP6). Loading doses of nusinersen were administered between TP1 and TP2 and the following maintenance doses corresponded to displayed time points (TP2 to TP6) 4 months apart. HFMSE, Hammersmith functional motor scale expanded; RULM, revised upper limb module; BL, baseline; TP, time point.

The overview of the longitudinal data of the aforementioned standardized scales is summarized in Figure 1. Since the beginning of therapy, all three patients showed a mild improvement in HFMSE scores in the initial period of observation, with a continuous positive trend being only shown for Patient 2. Patients 2 and 3 improved RULM scores at TP6, while Patient 1 remained stable. Additionally, Patient 2, who had a pre-treatment asymmetric strength deficit of the upper limbs (see Table 1), showed a bilateral improvement during the observation period (as revealed by the RULM scale). The performance of Patient 1 in 6MWT remained constant over time points.

### Muscle MRI

The image quality was reviewed by an experienced radiologist and positively evaluated for subsequent analysis.

All data for individual muscles and global ROIs are reported for TP1, TP3, and TP6 in the Supplementary Tables 1A,B, while Table 2 show the FF and w-T2 and their evolution over time for each compartment separately and globally (global ROI) at TP1 and at the end of the follow-up (TP6).

**Table 2:** Per-compartment quantitative muscle MRI measures.

	**Anterior compartment**	**Medial compartment**	**Posterior compartment**	**Global ROI**
(A) Average Fat fraction (FF) percentage for each compartment (anterior, medial, and posterior) and for the global ROI at baseline (TP1) and at the end of follow-up (TP6). Left and right sides are averaged. The increase/decrease (delta) is reported as percentage.				
TP1	51.2	54.3	45.8	50
TP6	57.1	57.1	54.5	57
Delta	+10.9%	+5.1%	+19.8%	+14%
(B) Average w-T2 in ms for each compartment (anterior, medial, and posterior) and for the global ROI at baseline (TP1) and at the end of the follow-up (TP6). Left and right sides are averaged. The increase/decrease (delta) is reported as percentage.				
TP1	43.7	44.4	30.4	43.73
TP6	42.6	41.1	28.1	41.68
Delta	−2.4%	−7.2%	−0.6%	−4.7%

**Figure 2:**
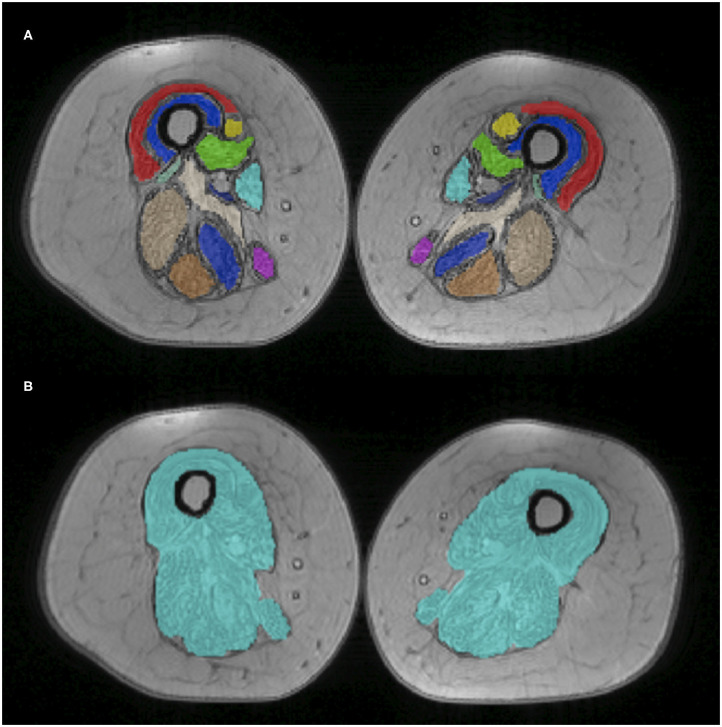
Thigh muscle regions of interest. Example of manually drawn muscle ROIs at middle thigh level, superimposed on the T2 GRE sequence (Patient 2 at TP1). On top **(A)**, the 12 muscle ROIs are individually displayed with different colors; at the bottom **(B)**, the global ROI comprising all muscles is shown. The 12 ROIs include VL, vastus lateralis; VM, vastus medialis; VI, vastus intermedius; RF, rectus femoralis; S, Sartorius; G, gracilis; AM, adductor magnus; AL, adductor longus; SM, semimembranous; SM, semitendinosus; BFL, biceps femoris longus head.

The average value of FF across the three SMA patients evaluated with a single ROI (see Figure 2) was 50% at TP1 and increased to 56.7% at the end of the follow-up (TP6). All muscles showed progression of FF with the only exception of the adductor longus (AL), which showed a reduction of FF from 39 to 37% (−6.02%) at TP6 (Supplementary Table 1A). For this reason, we decided to exclude AL from the analysis of the medial compartment and considered it on its own. In the posterior compartment, the most involved muscle was the semitendinosus (ST) (0.47% FF at TP1), which also progressed to 0.77% at TP6.

For the anterior compartment, FF progressed from 51.2 to 57.1% (average increase of +10.87%); for the medial compartment (excluding AL), from 54.3 to 57.1% (+5.12%); and for the posterior compartment, from 45.8 to 54.5% (+19.81%).

For what concerns the evaluation of w-T2, we found that w-T2 on the global ROI was 43.73 ms at TP1 (the higher value being represented by the medial compartment), decreasing over time (−4.7% in average at TP6 compared to TP1). All muscles showed a decrease of w-T2 over time (from TP1 to TP6), with the exception of AL, which showed an increase over time of 6% (from 43.84 to 45.11 ms) (Supplementary Table 1B).

### SC MRI

#### Dataset and Image Quality

**Figure 3:**
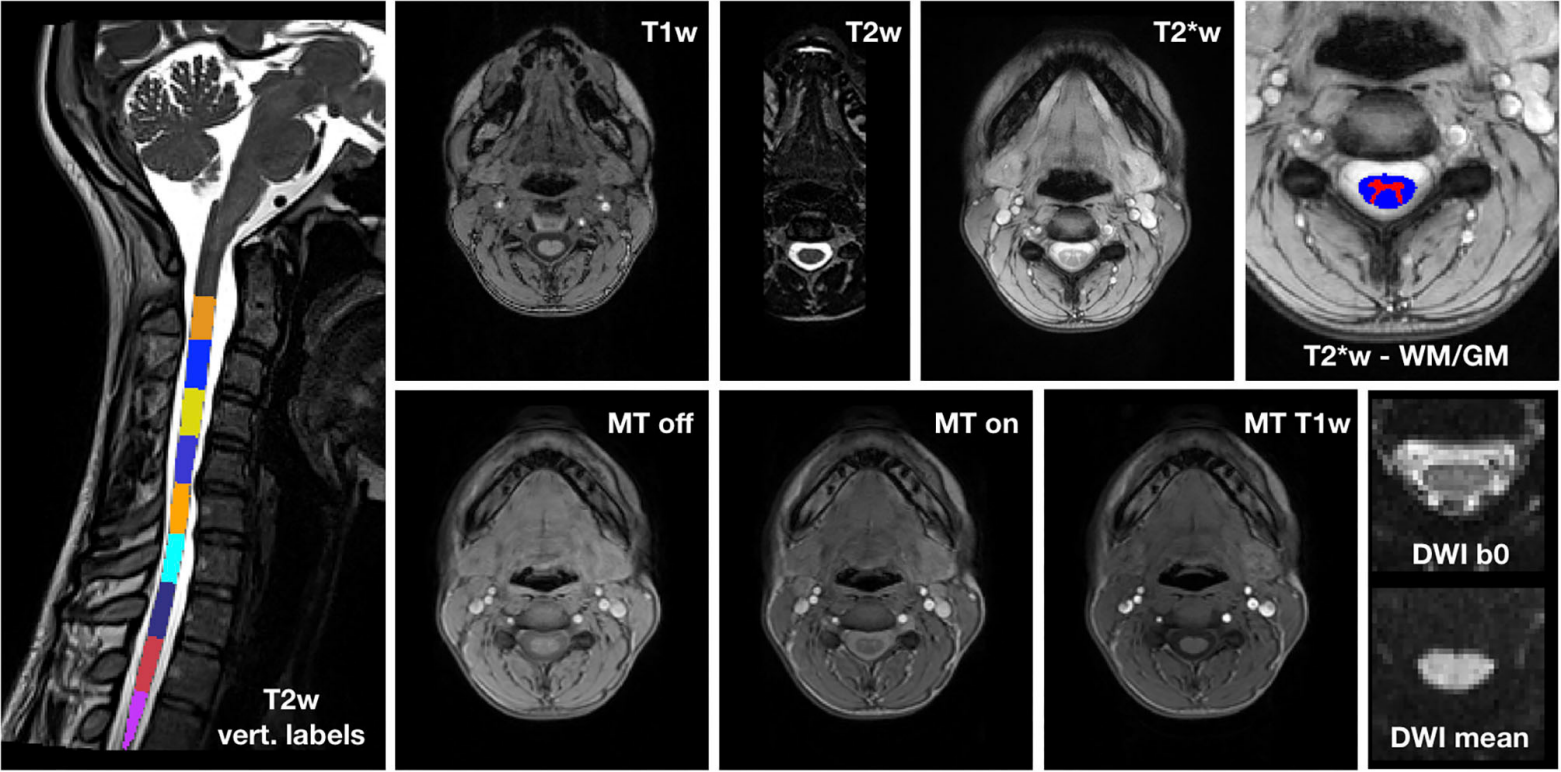
Exemplary spinal cord MRI dataset. On the left, T2w image with vertebral labels from C2 to D3. Top row: T1w image; T2w image; T2*w image; detail of T2*w image with WM and GM segmentation masks. Bottom row: MT (MT-off, MT-on and MT-T1w) and DWI (mean b0 image and mean DWI) dataset.

Images from an exemplary dataset of a randomly selected HC are shown in Figure 3.

The image quality was overall very good. Where motion artifacts and loss of signal and contrast in 3D T1w and T2w images were detected, we interrupted segmentation and vertebral labeling below their D1 and D2 levels, respectively, for one HC, and below C6 for Patient 3 at TP5.

#### Cross-Sectional Analysis of SC

**Figure 4:**
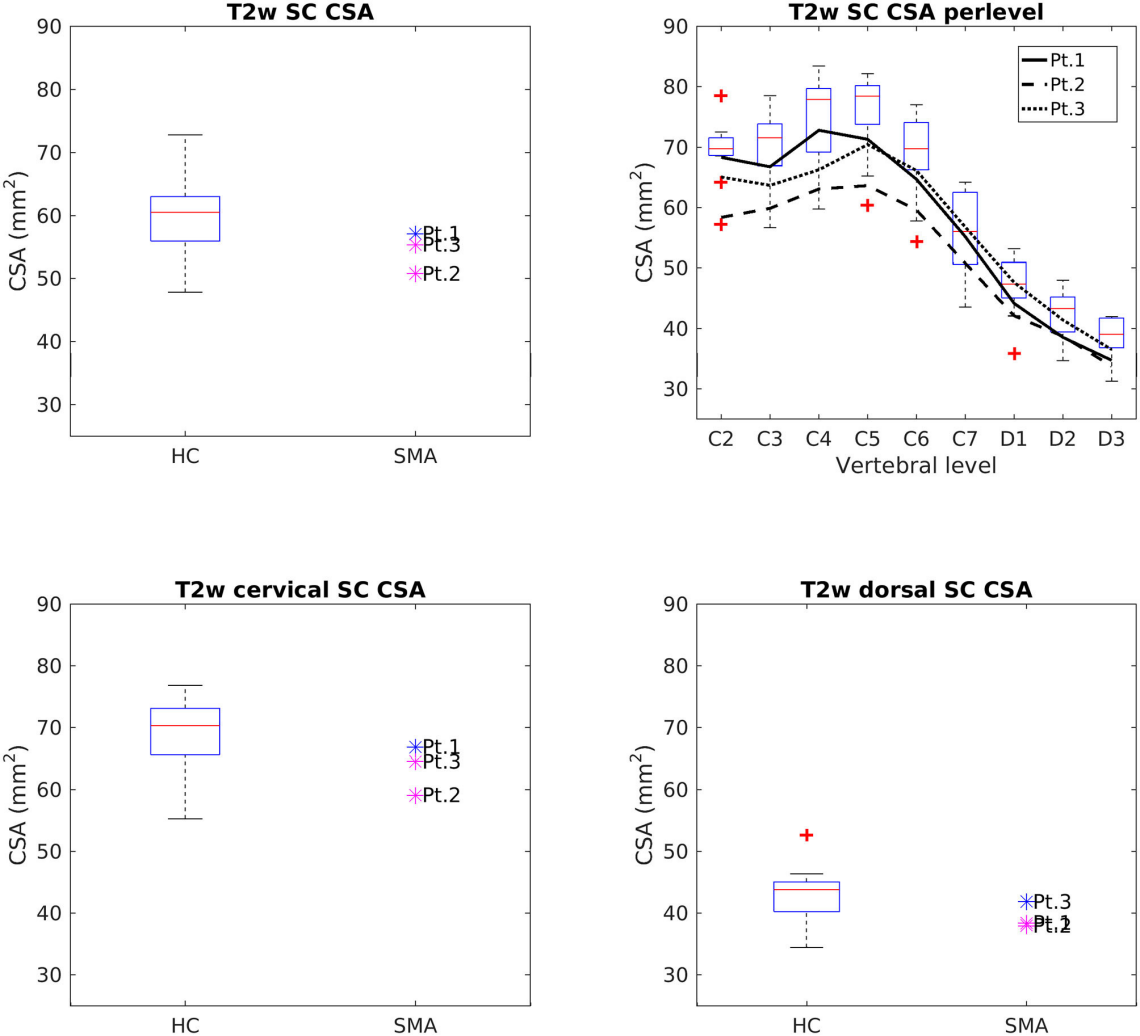
Cross-sectional comparison of CSA values measured on T2w images at TP3. Top left: average SC CSA from C2 to D3 in HC (box on the left) and SMA patients (dots on the right). Top right: per-level SC CSA values from C2 to D3 in HC (boxes) and SMA patients (black lines). Bottom left: average SC CSA from C2 to C7. Bottom right: average SC CSA from D1 to D3. The color of patient dots relates to their position compared to the respective distribution of HC values: blue for patient values within the 25–75th percentile range, magenta for patient values outside the 25–75th percentile range but within the HC distribution, red for patient values outside the HC distribution. Red + represent outlier HCs.

Figure 4 reports group results of CSA values evaluated on T2w images of HC and SMA subjects acquired at TP3. We found that SC CSA mean values of SMA patients fall in the lower range of the HC distribution, with two patients lying below the 25th percentile. The “per level” analysis additionally showed that the difference between the two groups was more pronounced at the cervical level with respect to upper dorsal vertebrae, even if the cervical intra-group variability is higher than the dorsal one for both groups; the difference between HC and SMA is maximum around from C3 to C6. SC CSA values computed on T2^*^w and T1w images confirmed this result.

**Figure 5:**
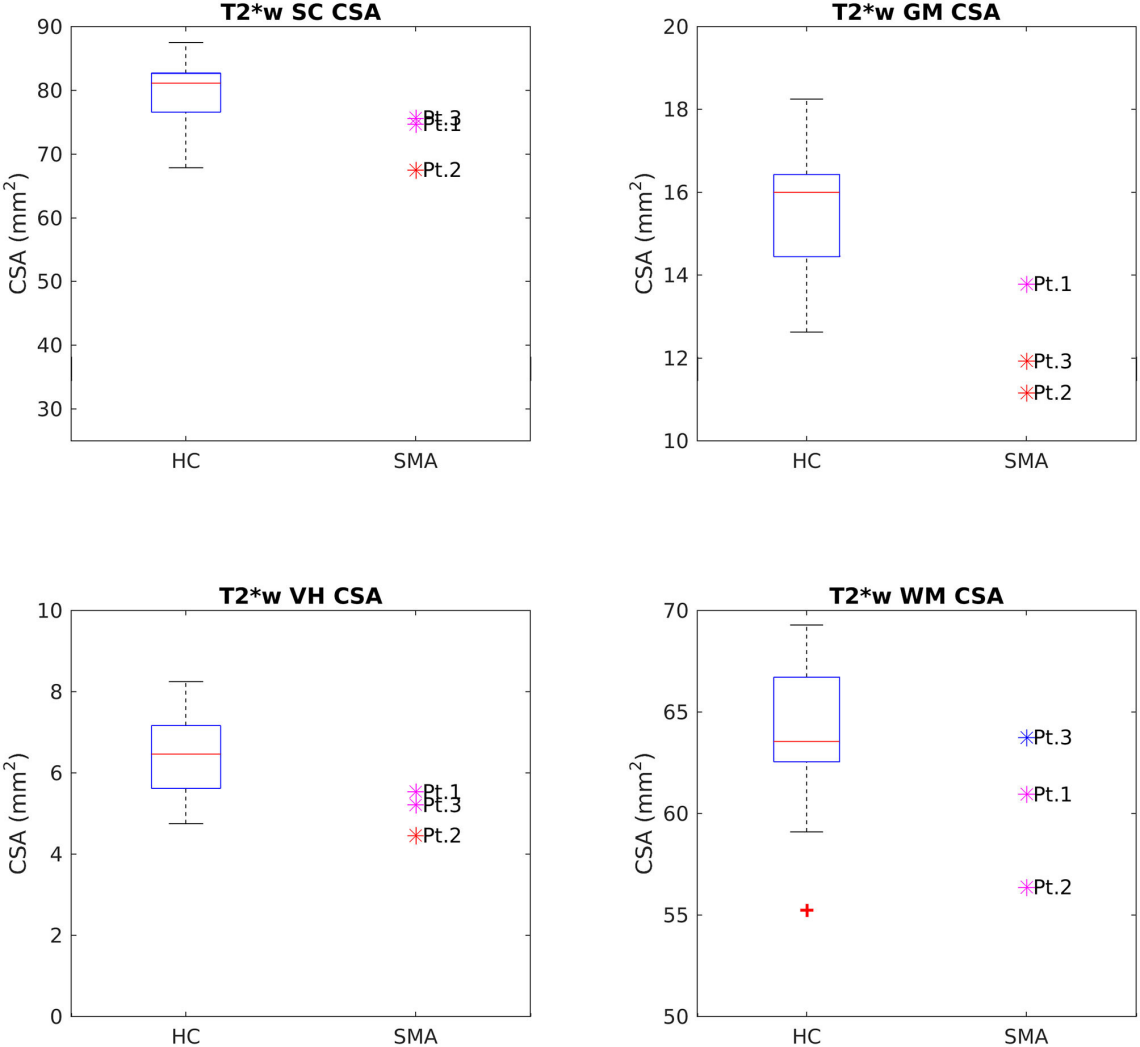
Cross-sectional comparison of CSA values measured on T2*w images at TP3. From left to right and top to bottom: average CSA values of SC, GM, VHs, and WM measured from C3 to C4 in HC (box on the left) and SMA patients (dots on the right). The color coding of patient dots is explained in the caption of Figure 4. Red + represent outlier HCs.

CSA of GM and VHs analyzed on T2^*^w images were reduced in patients compared to HC. No noticeable asymmetry was found between the right and left sides of GM and VHs (see Figure 5).

**Figure 6:**
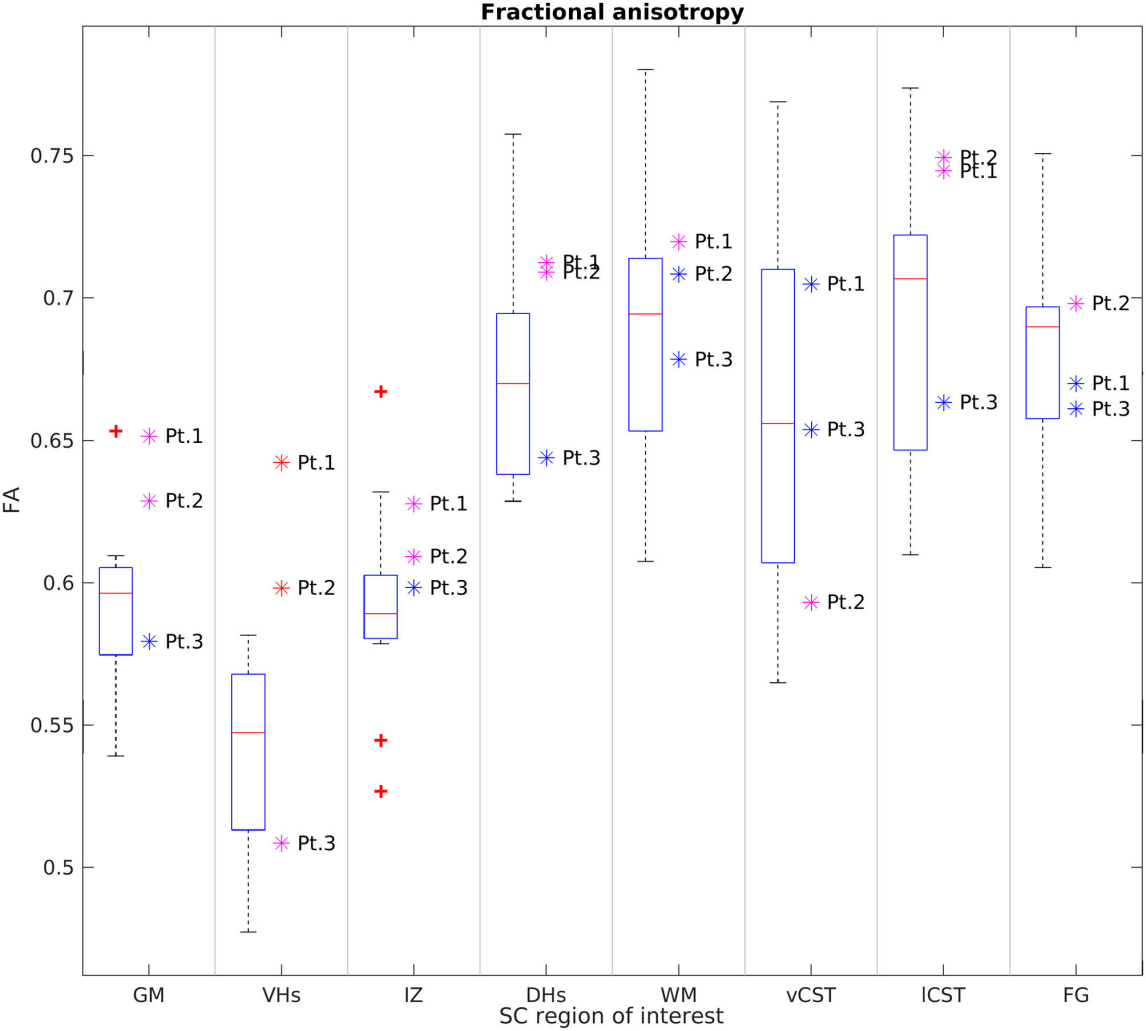
Cross-sectional comparison of average FA values measured at TP3. From left to right: average FA values of GM, VHs, IZ, DHs, WM, vCST, lCST, and FG measured from C3 to C4 in HC (box on the left) and SMA patients (dots on the right). The color coding of patient dots is explained in the caption of Figure 4. Red + represent outlier HCs.

**Figure 7:**
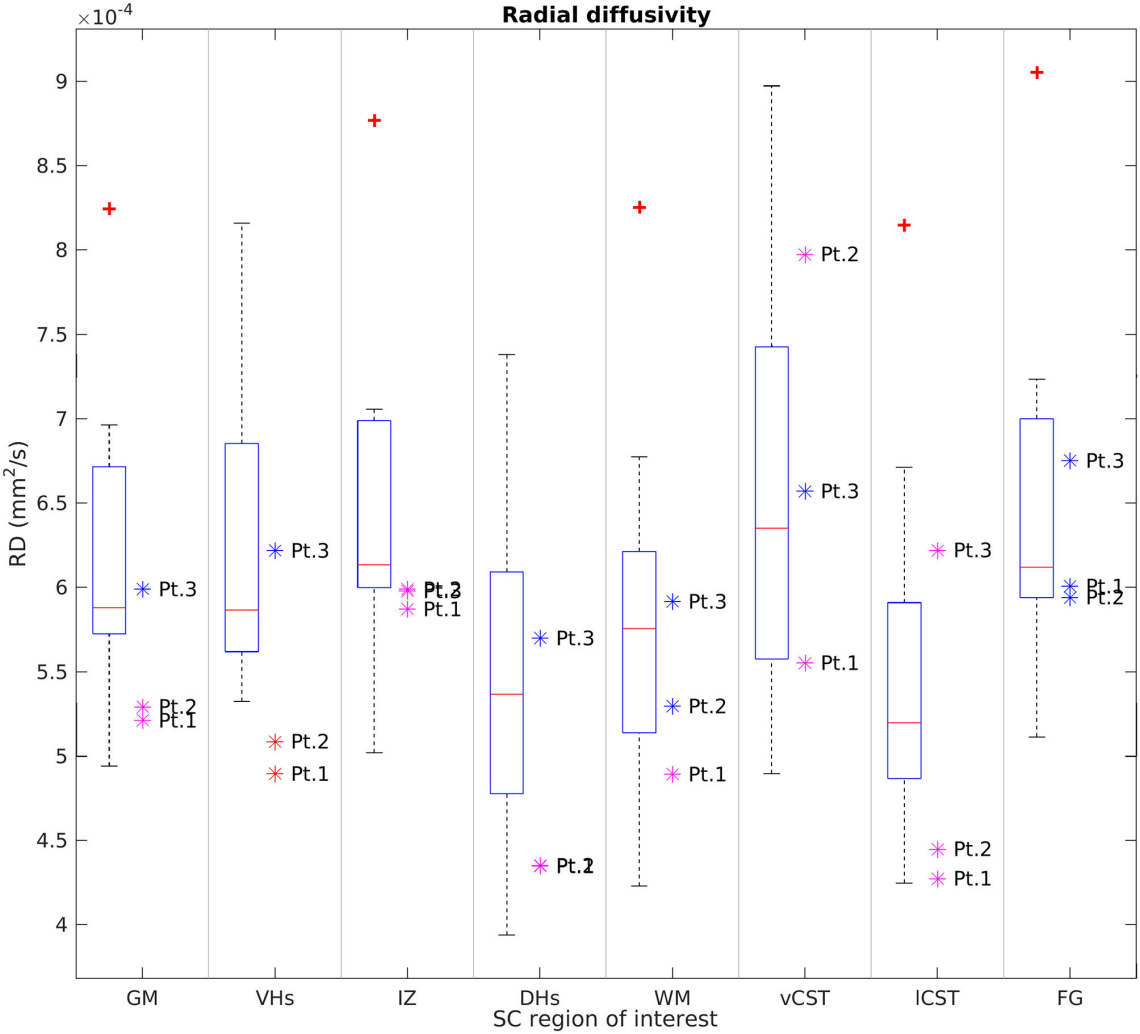
Cross-sectional comparison of average RD values measured at TP3. From left to right: average RD values of GM, VHs, IZ, DHs, WM, vCST, lCST, and FG measured from C3 to C4 in HC (box on the left) and SMA patients (dots on the right). The color coding of patient dots is explained in the caption of Figure 4. Red + represent outlier HCs.

Figures 6, 7 report group results of DTI measures (FA and RD, respectively) for HC and SMA subjects at TP3. Analogous plots for AD and MD can be found in Supplementary Figures 1, 2. In GM, we report a reduction in RD and an increase in FA in SMA patients with respect to HC, while no differences can be observed for AD and MD values. Accordingly, FA and RD values of the VHs of two out of three SMA patients fall outside the range of the corresponding HC distribution, while MD and AD of the VHs differ from the range of HC to a lesser extent.

We found no univocal variation in WM and that DTI values of most WM regions in SMA patients fell within the range of the respective HC distribution.

**Figure 8:**
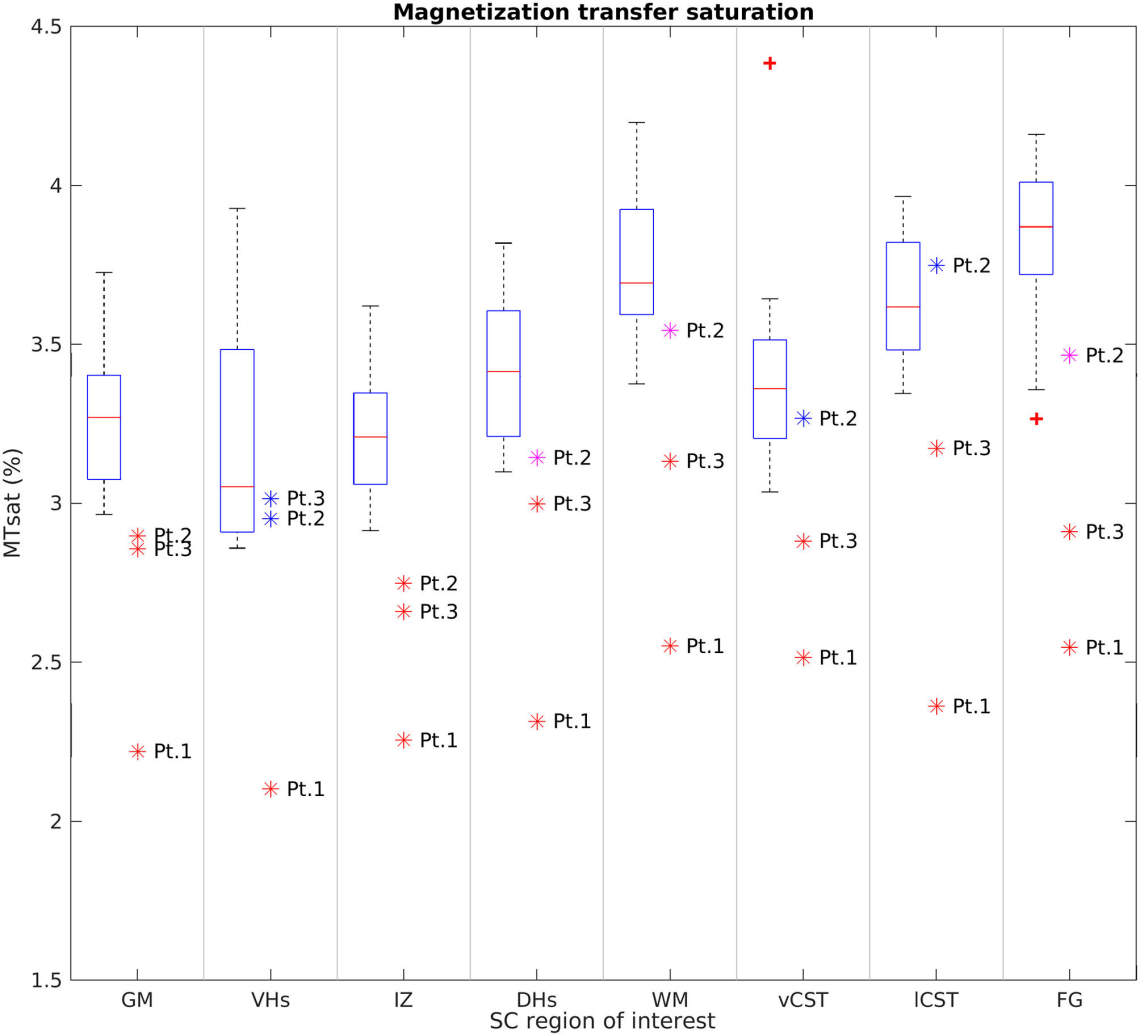
Cross-sectional comparison of average MTsat values measured at TP3. From left to right: average MTsat values of GM, VHs, IZ, DHs, WM, vCST, lCST, and FG measured from C3 to C4 in HC (box on the left) and SMA patients (dots on the right). The color coding of patient dots is explained in the caption of Figure 4. Red + represent outlier HCs.

Figure 8 reports group results obtained from MTsat maps of HC and SMA patients at TP3. We found a decrease of MTsat values in both WM and GM of SMA patients with respect to the HC group. Moreover, we found an evident decrease of MTsat average values in the specific investigated WM bundles (vCST, lCST, and FG). In particular, we found that the MTsat values of two patients out of three fell always outside the corresponding HC group range of values for each of these regions. In GM, the reduction of MTsat is more pronounced in the intermediate regions of the GM, with respect to ventral or dorsal horns.

#### Longitudinal Analysis of SC

**Figure 9:**
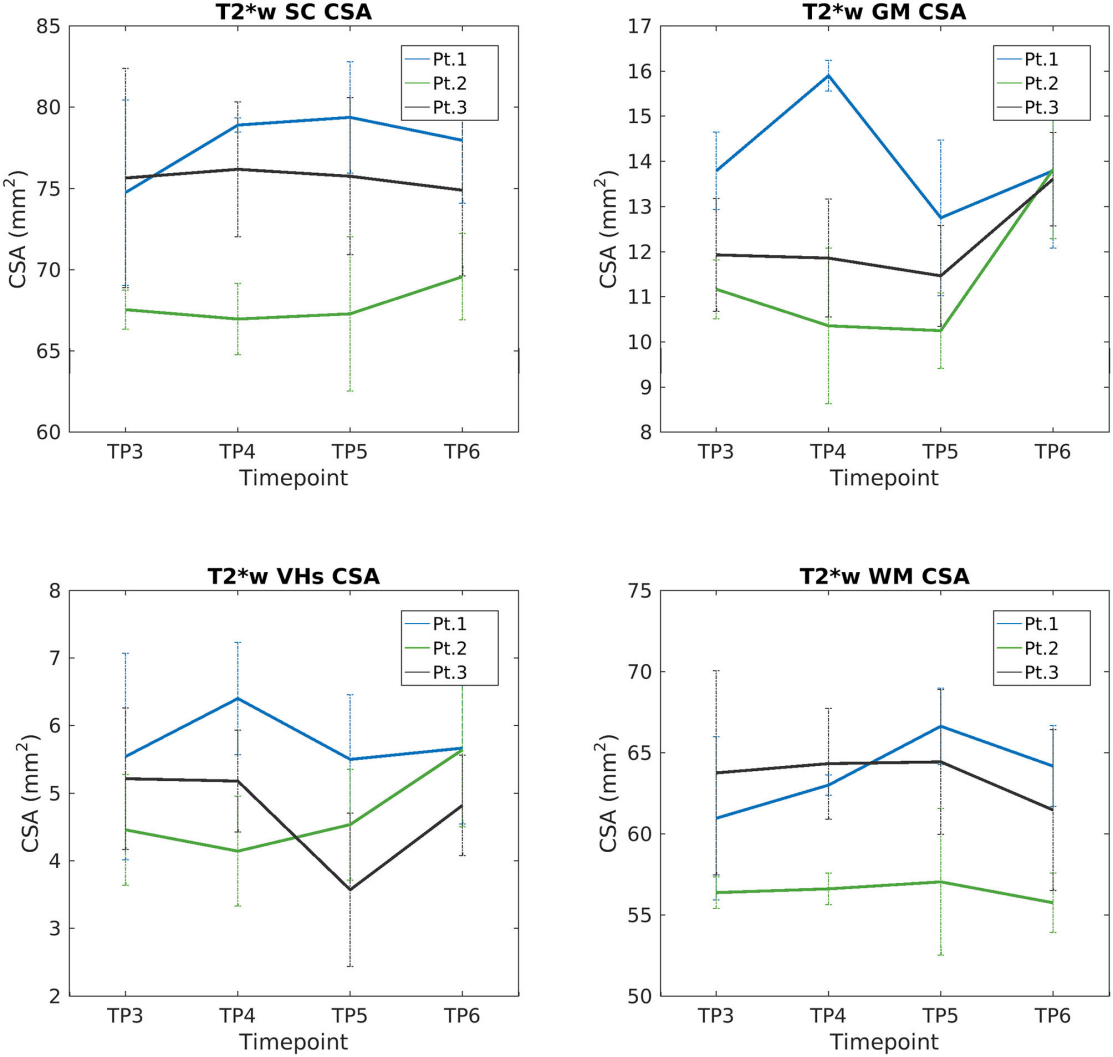
Longitudinal analysis of average CSA values measured on T2*w images. From left to right and top to bottom: average CSA values of SC, GM, VHs, and WM measured from C3 to C4 starting from TP3 to TP6.

**Figure 10:**
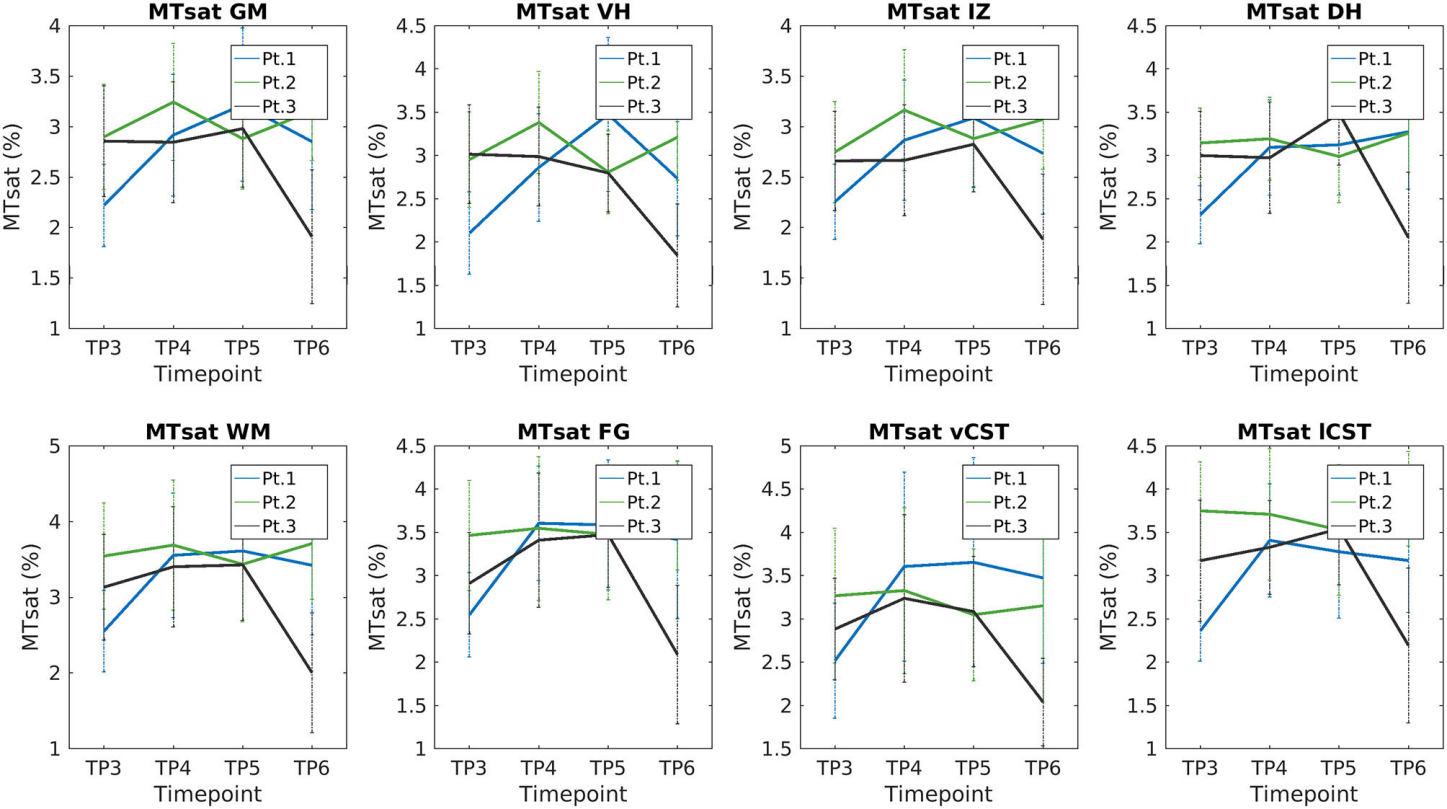
Longitudinal analysis of average MTsat values. From left to right and top to bottom: average MTsat values of GM, VHs, IZ, DHs, WM, vCST, lCST, and FG measured from C3 to C4 starting from TP3 to TP6.

We report a trend of increasing SC, GM, and VHs CSA values in T2^*^w images of SMA Patient 2. CSA values of Patients 1 and 3 do not show evident trends over time (see Figure 9). Mean DTI metrics computed do not show evident trends over time and they are consistent between patients (see Supplementary Figures 3–6). The longitudinal trend observed for MTsat values shows great similarities between different ROIs. In particular, this longitudinal analysis highlighted a substantially stable pattern of MTsat in each ROI of Patient 2. On the other hand, data from Patient 1 showed a remarkable increase from TP3 to TP4, while Patient 3 exhibited an abrupt drop from TP5 to TP6 (see Figure 10).

## Discussion

This pilot study applied qMRI to measure changes in muscle and cervical SC of three adult Type 3a SMA patients under nusinersen treatment with a longitudinal follow-up of 21 months, assessing muscle and SC degeneration, with the aim to evaluate whether MRI could provide useful biomarkers to monitor the clinical efficacy of nusinersen. Patients were clinically stable over the course of this study, while muscle qMRI suggested the presence of fat substitution progression and w-T2 slight decrease over time in thigh muscles. In the SC, we observed reduced CSA, GM, and VHs CSA of SMA patients transversally compared to HCs at TP3; these values remained generally stable over time, although Patient 2 exhibited a trend of increasing GM and VHs area over time. Diffusion parameters were altered when compared to HCs, but stable over time in SMA subjects, while MTsat values of SC structures were also reduced.

### Clinical Data

Clinically, all enrolled SMA Type 3a patients were young adult females with similar features, characterized by the early onset of a typical clinical picture of SMA, and a slow progression.

The three patients reported a subjective general improvement as to their motor performance and “self-confidence” during the study; none of them experienced any side effect during treatment. This subjective perception of improvement in motor performance reported by patients was also confirmed by the results of validated motor function tests: all patients obtained an improvement in the HMFSE scale from TP1 to TP2, when they reached a plateau. Patients 2 and 3 exhibited also an improvement in the RULM score at TP6, while the score of Patient 1 remained stable. This could be due to a general better motor function of Patient 1, who had a very high score already at the baseline (TP1). Furthermore, Patient 2, who had developed asymmetric weakness in upper limb scores before the treatment, showed a bilateral good recovery, but asymmetry essentially remained identical. The normal cervical MRI (no signs of compression or plexopathy were found) and the absence of both a clinical history of trauma and other neurological signs suggest that such asymmetry could be considered as an atypical manifestation of the disease in this patient. Even though SMA is generally described as a symmetric disorder, it is not uncommon to see asymmetrical weakness: Kang et al. (46) reported three female type 3 SMA patients with asymmetrical weakness, showing also upper motor neuron signs that have always been absent in our patient. Despite the small sample size considered here and the relatively short observation period, our results are in line with those reported by the German and Italian studies (47, 48). It should also be noted that all patients reported some further improvement in motor function, which could not be captured by their repeated functional scores possibly because of the discrete nature of such scales.

### Muscle MRI

In all three patients, a progression of thigh muscle fat substitution was documented over time (mean increase of roughly 7% in 21 months when considering the global cross-sectional muscle ROI). This progressive fat substitution can represent the natural evolution of the disease over time, despite the concomitant therapy and the global stability of clinical scales. Unfortunately, to our knowledge, no longitudinal quantitative muscle MRI data on the natural history of the disease are available for comparison at present. The posterior compartment of the thigh showed the greatest percentage increase in FF (+19.81%) in our cohort, but the most elevated fat replacement was shown in the anterior and medial compartments (both with 57.1% FF at the end of follow-up, TP6), the medial compartment being the most involved from the beginning of the study. This anterior compartment predominant involvement is in line with other qualitative and quantitative muscle MRI studies conducted so far, including the relative sparing of the adductor longus (AL) (26, 39). We also confirmed the greater fat replacement of the semitendinosus (ST) among other hamstring muscles, already demonstrated semiquantitatively by Brogna et al. (39). By contrast, the relative sparing of the medial compartment previously demonstrated semiquantitatively by Brogna and colleagues was not confirmed by the present quantitative data, possibly because we did not perform a muscle CSA analysis.

Given the small number of cases included in this pilot study, we did not perform any correlation analysis between quantitative muscle features and clinical scales, neither at TP1 nor at the end of follow-up. Even considering the global stability of the disease as observed with clinical scores, FF quantification was able to detect subtle changes in the muscle, thus once again demonstrating the potential of applying fat replacement quantification as a surrogate outcome measure for clinical trials.

FF, however, represents only part of the disease process, becoming evident at advanced stages pathology. By contrast, high w-T2 signal has been proposed in neuromuscular disorder studies as a potential marker of disease activity, though non-specific, mostly reflecting initial stages of the disease (15).

Previous studies exploring w-T2 in thigh muscles in SMA patients reported an increased value in thigh muscles (49, 50) compared to HCs. Bonati et al. (49) obtained w-T2 values exceeding 60 ms in SMA muscles, but used a mono-exponential fit for the T2w multi-echo sequence. Chabanon et al. (50) applied a multi-exponential signal model and found increased w-T2 values (ranging from 34.3 to 31.3 ms), but did not take into account EPG fitting. By contrast, Otto et al. (20) adopted a multi-exponential model accounting for EPG fitting and did not report values higher than 31 ms at most in SMA, thus suggesting that differences in values could be mostly due to methodological issues and not to pathological mechanisms.

In the present longitudinal study, we also applied multicompartment EPG fitting for T2 mapping and we found thigh muscle w-T2 ranging from 39.66 to 52.64 ms in SMA patients (average 43.73 ±3.73 ms at TP1, before the start of therapy), values that are slightly high when compared to previously reported values (20, 49). Additionally, w-T2 seemed to slightly decrease over time, with the greatest percentage reduction in the medial compartment at the last time point (TP6, 21 months after the beginning of therapy). As already reported by Otto et al. (20), such data have to be considered cautiously and we agree with the authors that artifacts or methodological issues may have a great impact in evaluating w-T2 in highly fat-replaced muscles. In addition to this, we know from the literature that w-T2 values are quite variable and linked to a number of physiological and paraphysiological conditions (in addition to the pathological ones) (15, 51). However, we still cannot say whether such abnormalities are due to the natural evolution of muscle pathology in SMA or therapy-induced. Longitudinal future studies applying quantitative muscle MRI to evaluate w-T2 with optimized methodological post-processing and performed on larger cohorts may help to address the question.

DTI has recently been applied as a supplementary advanced muscle MRI technique to evaluate microstructural changes in muscle in SMA: Barp et al. (19) found a reduced fractional anisotropy (FA) over time during nusinersen treatment (24 months follow-up) in two patients, whereas Otto et al. (20) found an increase in FA (and a decrease in MD) in SMA compared to HCs. DTI studies of thigh muscle may help to investigate muscle microstructural properties in SMA beyond FF and w-T2, as already suggested (20).

### Cervical SC MRI

#### Cross-Sectional Analysis of SC

We found a clear difference in total CSA of the SC between SMA patients and HCs at TP3, in substantial agreement with previous studies (27–29). SMA patients showed a smaller CSA than HCs, with the largest discrepancy being observed in correspondence of the cervical vertebrae. Such finding is consistent with the typical limb girdle weakness of type 3 SMA. This result was confirmed by segmentations obtained from both T1w and T2w volumetric sequences; the difference in the absolute values of CSA from the two methods can be explained by the different spatial resolution of the two acquisitions. SC GM was segmented in high in-plane resolution T2^*^w sequences between C2 and C4 and SMA patients showed a reduced area with respect to HCs, thus confirming a pathology-related GM atrophy. The VHs contain the motor neurons population of the SC and, therefore, their shrinkage could reflect specific αMNs degeneration (12). However, given the small size of these structures in relation to the MRI resolution, this result could be affected by partial volume and segmentation errors and should therefore be interpreted with caution.

Even though SMA is generally described as a symmetric disorder, in clinical practice, it is not uncommon to see asymmetrical weakness. In this case, the analysis that we performed to investigate the asymmetry of clinical worsening observed in Patient 2 before beginning treatment produced no evidence of altered qMRI parameters between the two sides that could explain it.

Our measurements of the CSA of SC, GM, and VH are in line with the known degeneration of αMN caused by SMA. In agreement with previous studies (28, 29), we did not detect pathological alterations of diffusion parameters in the WM of SMA patients. Conversely, we documented an increase of GM FA and a slight reduction of RD, while AD and MD were unchanged. In particular, a similar FA variation was also found in VH ROIs. As pointed out also by Stam et al. (29), resolution-driven partial volume effects due to the inclusion of adjacent WM regions could be a possible cause of altered average FA values, because such effects can be further enhanced by GM shrinkage induced by pathology. However, partial volume effects should be limited by the use of probabilistic labels in the averaging operation. Furthermore, if such changes were due to only partial volume, we should also expect altered MD and AD values because DTI maps are inherently co-registered. Therefore, another possible explanation is that motor neuron degeneration, together with the consequent GM shrinkage, enhances the relative volume fraction of those WM fibers running through the SC GM, thus increasing the resulting FA. A third possibility is that we are observing the effects of gliosis, which was reported to occur in the VH of SMA patients (52). Given the low number of literature histopathological studies, these hypotheses should only be regarded as speculative. Future *in vivo* and *ex vivo* diffusion studies, conducted with newly developed high resolution DWI techniques and fitted with advanced diffusion models, together with concomitant histological studies, will certainly help to better discriminate between these scenarios.

To further understand the microstructural modifications underlying SMA, we acquired MT-weighted images. Changes in MTsat values are considered an indirect marker of changes in myelin content (45, 53). We found a remarkable reduction of MTsat values in both WM and GM, suggesting a loss of myelinated fibers. The reduced MTsat values observed in FG are in agreement with previous neuropathological studies of SMA, which consistently reported a loss of myelinated fibers in such tracts (52, 54, 55). In addition, myelin loss was also occasionally found to occur in the lateral columns (55, 56) and MTsat values from the corticospinal tracts of SMA patients confirm this. The alteration of MTsat values in SC GM could be explained by partial volume between GM regions and the abovementioned WM tracts; this hypothesis is supported by the fact that the most pronounced reduction of MTsat values was found in the intermediate region of the GM, which is the closest to the FG, the tract that was most frequently reported to be severely affected by demyelination. To the best of our knowledge, this is the first time that MT imaging is applied to investigate SC myelin content in SMA *in vivo*. Given the encouraging agreement between our results and those reported in neuropathological studies, MT measures certainly represent promising candidates for better understanding and monitoring the evolution of SMA.

#### Longitudinal Analysis of SC

The longitudinal evaluation of CSA and GM over time showed stable values for two out of three patients, while Patient 2 showed a slight increase in SC, GM, and VH CSA values. Therefore, the SC volume can be considered stable over 1 year of disease and treatment monitoring. We can hypothesize that the relative volume stability of SC could represent a positive response to therapy: we do not have the pre-treatment quantitative volumetric data of the natural history of the disease and we do not have a control group of untreated SMA patients, yet we could assume that SC volumes (total, GM, and VHs) would have presumably shown a progressive reduction over time without treatment. Such hypothesis is in line with the stability of SC and GM CSA values over time that mirror the clinical improvement shown by increased HMFSE and RULM scores. From the histological point of view, an increased volume of the VH could be compatible with the effects of nusinersen on the motor neuron population, as suggested in a preclinical study in mice (57). The authors used choline acetyltransferase (ChAT, a marker of αMNs) to stain the SC αMNs in a murine model of SMA and reported that nusinersen could effectively increase the number of ChAT-positive cells by 38%, and up to 62%, with respect to untreated SMA mice, thus proving the recovery of a fraction of the degenerated cell population. This result was also confirmed by another group that “detected a recovery in the number and chemical composition of Cajal Bodies” in treated SMA patients, suggesting that the depletion process of Cajal Bodies found in motor neurons from the VH of untreated SMA mice models may be reversed by nusinersen (58). The number of Cajal Bodies is strongly bound to the cell body size (59); therefore, the high number of motor neurons involved in the degeneration/recovery processes and the histological findings reported above suggest that an enlargement of GM, and in particular of the VH areas, could be expected during nusinersen treatment; our results suggest that this effect could also be detected with advanced MRI techniques. It can be argued also that such changes in cell body size during the observation period could impact GM diffusion parameters and, in particular, MD. However, it should be considered that no changes in GM MD were detected between HC and SMA groups at TP3 and that the MD of SMA patients remained stable over time. Consequently, we could hypothesize that the degree of cellular swelling may not be sufficient to produce detectable effects on MD, but it is not excluded that higher-order diffusion models may be able to capture such subtle changes. Furthermore, we do not know exactly the time scale over which these changes may occur after beginning treatment. In this regard, it is essential that future studies closely monitor SC parameters before and during the administration of the loading dose of nusinersen.

### Limitations

In this study, we acknowledge a number of limitations. The first and most relevant is the small sample size of the cohort of enrolled patients that impacted our ability to perform statistical analysis. In addition to that, the study is penalized by the lack of a group of untreated SMA patients: this would have provided a benchmark to better quantify the effects of nusinersen on SC structures over the observation period. Second, we started muscle MRI at TP1, but SC at TP3, so we do not have a proper baseline for this technique. Moreover, it is also possible that 1 year is too short to detect possibly subtle, though significant, changes induced by therapy. To this end, we plan to recall the patients 1 year from the last MRI session to repeat the qMRI scan. These limitations prevented us from clearly assessing whether qMRI parameters can be considered valid biomarkers of disease evolution and treatment efficacy. Future studies should aim to enroll larger cohorts of patients for a longer observation period in order to further investigate the relationship between qMRI parameters and clinical scores through adequate statistical analysis and disease progression models. This could provide a valuable tool to better characterize the natural history of the disease and to predict its evolution in particular in relation to different therapeutic approaches.

A final consideration is that more advanced diffusion and myelin imaging techniques could provide more specific or sensitive biomarkers of pathology. However, we believe that the SC MRI protocol adopted here represents a good trade-off between clinical and research needs, thanks to its relatively short acquisition time in relation to the high number and variety of techniques involved, which allowed us to investigate several aspects of the SC (micro-)structure. On the basis of the findings reported here, it will be possible to make informed choices to better address more specific questions and challenges posed by the disease.

## Conclusions

In this pilot MRI study, we demonstrated a progression of fat substitution in thigh muscles of SMA 3a patients during therapy and a concomitant trend toward a slight reduction of w-T2. The analysis of SC data confirmed a degeneration of tissues highlighted by both GM atrophy and, interestingly, reduced MTsat values, the latter of which have never been reported in SMA before. Our data warrant future studies to further investigate whether qMRI parameters may represent valid biomarkers of pathology and treatment efficacy in SMA, but this will require longer longitudinal studies on larger cohorts and proper statistical models of disease progression.

## Data Availability Statement

The datasets presented in this article are not readily available because of institutional policies. Requests to access the datasets should be directed to anna.pichiecchio@mondino.it.

## Ethics Statement

The studies involving human participants were reviewed and approved by Comitato Etico Area Referente Pavia della Fondazione IRCCS Policlinico San Matteo. The patients/participants provided their written informed consent to participate in this study.

## Author Contributions

AP, CG, GS, and MP contributed to the conception and design of the work. GS, FSa, and XD set up the acquisition protocol. GS, FSo, MP, AP, and GG acquired the data. GS, CA, FSo, NB, and SM performed data analysis. GS, AB, CG, SP, AP, MP, and EP contributed to the interpretation of results. AB, AG, SP, and EP performed clinical evaluation of patients. LF, AP, and SB administered the treatment. GS, CA, MP, and AP wrote the first draft of the manuscript. All authors contributed to manuscript critical revision, read, and approved the submitted manuscript.

## Conflict of Interest

AP is a member of the advisory board and consultant for G-enzyme Sanofi. The remaining authors declare that the research was conducted in the absence of any commercial or financial relationships that could be construed as a potential conflict of interest.

## Abbreviations

6MWT: 6-minute walk test
AD: axial diffusivity
aMNs: alpha motor neurons
BL: baseline
C-MAP: compound muscle action potential
CSA: cross-sectional area
DHs: dorsal horns
DTI: diffusion tensor imaging
EPG: extended phase graph
FA: fractional anisotropy
FF: fat fraction
FG: fasciculus gracilis
GM: gray matter
HC: healthy controls
HFMSE: Hammersmith functional motor scale expanded
IZ: intermediate zone
lCST: lateral corticospinal tract
MD: mean diffusivity
MRI: magnetic resonance imaging
MT: magnetization transfer
MTsat: magnetization transfer saturation
qMRI: quantitative MRI
RD: radial diffusivity
ROIs: regions of interest
RULM: revised upper limb module
SC: spinal cord
SMA: spinal muscular atrophy
SMN1: survival motor neuron gene 1
T1w: T1-weighted
T2^*^w: T2^*^-weighted
T2w: T2-weighted
TP#: time point #
vCST: ventral corticospinal tract
VHs: ventral horns
w-T2: water T2
WM: white matter.
