# Supplementary material for: Pilot Study on Quantitative Cervical Cord and Muscular MRI in Spinal Muscular Atrophy: Promising Biomarkers of Disease Evolution and Treatment?
Source: Front Neurol. 2021 Mar 29;12:613834. doi: 10.3389/fneur.2021.613834 (PMC8039452; doi:10.3389/fneur.2021.613834)
Supplement: Supplementary file 1 [file Data_Sheet_1.pdf]

Supplementary Material

1 Supplementary Figures and Tables

Supplementary Table 1: Average muscle fat fraction and water-T2

Fat fraction (2A) and water-T2 (2B) values at each time-point are reported for the global ROI and for each considered individual muscle separately. For FF (2A), values are reported as percentage, w-T2 (2B) are expressed in ms. The last three lines report the average value for each time point between the subjects. Reported values are averaged between the left and right side.
VL = vastus lateralis, VM = vastus medialis, VI = vastus intermedius, RF = rectus femoralis, S = sartorius, G = gracilis, AM = adductor magnus, AL = adductor longus, SM = semimembranous, SM = semitendinosus, BFL = long head of biceps femoris.

Table 1A - FF

			Anterior compartment				Medial compartment				Posterior compartment		
**Patient**	**Timepoint**	**GLOBAL ROI**	**VL**	**VM**	**VI**	**RF**	**S**	**G**	**AM**	**AL**	**SM**	**ST**	**BFL**
1	TP1	0,53	0,70	0,33	0,44	0,58	0,85	0,77	0,57	0,66	0,45	0,47	0,38
	TP3	0,56	0,70	0,36	0,48	0,63	0,88	0,81	0,59	0,47	0,51	0,52	0,42
	TP6	0,61	0,68	0,35	0,50	0,71	0,91	0,73	0,56	0,47	0,59	0,77	0,37
2	TP1	0,41	0,72	0,27	0,43	0,43	0,34	0,52	0,34	0,17	0,23	0,35	0,37
	TP3	0,47	0,77	0,39	0,59	0,69	0,33	0,52	0,42	0,24	0,30	0,33	0,42
	TP6	0,51	0,82	0,39	0,52	0,58	0,45	0,58	0,36	0,25	0,33	0,45	0,42
3	TP1	0,57	0,73	0,36	0,58	0,59	0,54	0,41	0,55	0,36	0,67	0,57	0,61
	TP3	0,57	0,79	0,35	0,55	0,62	0,48	0,47	0,53	0,35	0,71	0,60	0,60
	TP6	0,59	0,75	0,40	0,56	0,59	0,54	0,47	0,53	0,39	0,72	0,63	0,62
Avg	TP1	0,50	0,72	0,32	0,48	0,54	0,58	0,57	0,48	0,39	0,45	0,46	0,45
	TP3	0,53	0,75	0,36	0,54	0,65	0,56	0,60	0,51	0,36	0,51	0,48	0,48
	TP6	0,57	0,75	0,38	0,53	0,63	0,63	0,59	0,49	0,37	0,55	0,62	0,47

Table 1B - w-T2

Patient	Timepoint	GLOBAL ROI	Anterior compartment				Medial compartment				Posterior compartment		
			VL	VM	VI	RF	S	G	AM	AL	SM	ST	BFL
1	TP1	42,17	42,57	45,19	44,24	37,02	56,10	39,87	42,35	41,41	41,80	41,00	41,46
	TP3	44,27	43,25	44,84	45,68	43,26	34,14	38,95	45,30	41,73	40,28	42,38	46,60
	TP6	43,44	43,55	46,19	46,16	39,77	42,90	38,40	44,55	46,18	35,80	38,83	43,65
2	TP1	45,78	46,94	45,25	46,39	42,61	49,67	41,13	48,00	47,11	41,45	41,90	42,76
	TP3	45,49	44,38	49,83	47,19	42,40	50,79	45,70	47,25	47,38	40,92	41,45	41,64
	TP6	38,33	34,85	37,50	37,38	39,95	38,66	41,36	41,11	43,27	36,12	36,34	39,11
3	TP1	42,82	40,77	50,15	41,92	41,50	52,15	42,80	41,98	43,00	35,75	38,99	39,40
	TP3	42,17	40,23	44,93	44,07	42,74	51,12	40,95	39,77	43,21	37,02	42,39	40,37
	TP6	43,24	44,15	47,76	50,42	43,41	58,50	45,76	36,70	45,87	33,56	37,74	35,53
Avg	TP1	43,73	43,42	46,86	44,18	40,38	52,64	41,27	44,11	43,84	39,66	40,63	41,20
	TP3	43,98	42,62	46,53	45,65	42,80	45,35	41,86	44,10	44,11	39,41	42,08	42,87
	TP6	41,68	40,85	43,82	44,65	41,04	46,69	41,84	40,79	45,11	35,16	37,64	39,43

1.1 Supplementary Figures

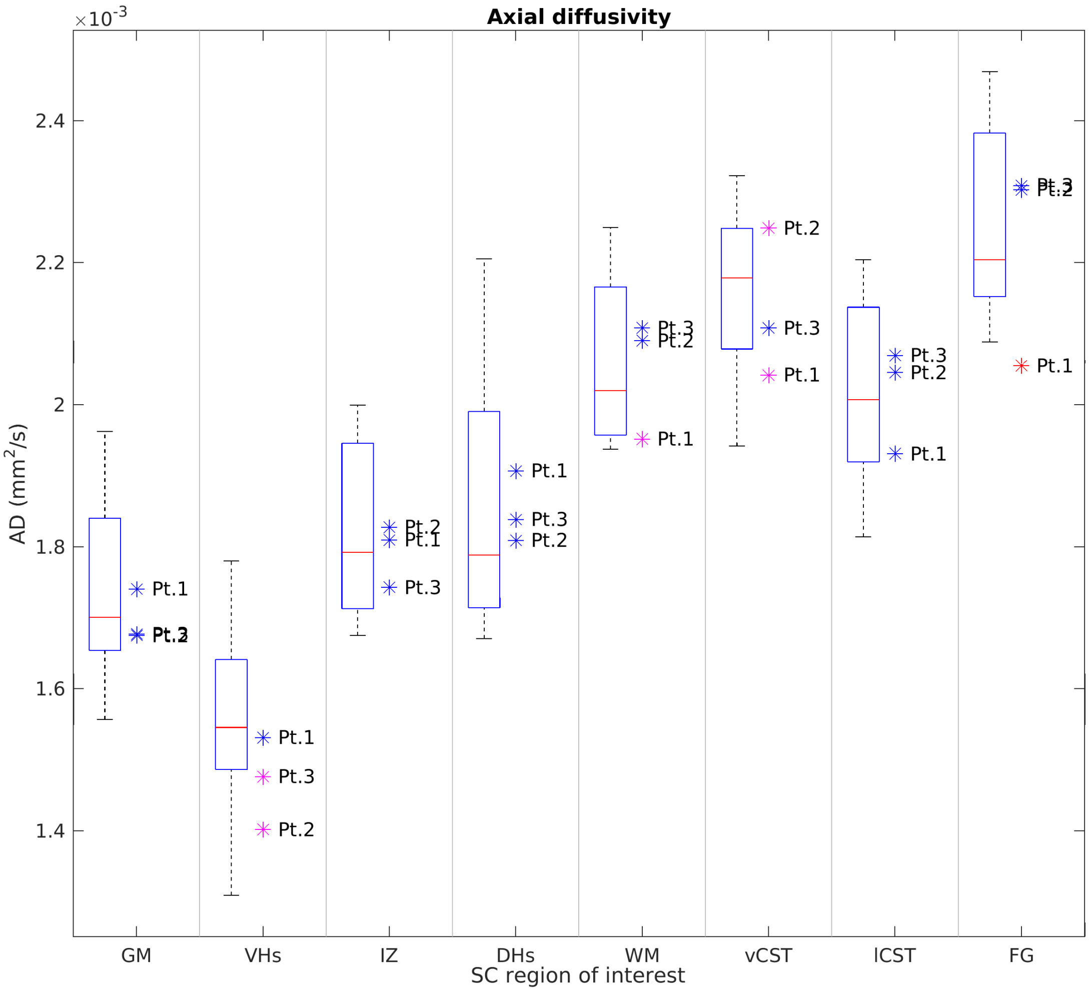

Supplementary Figure 1 - Cross-sectional comparison of average AD values measured at TP3. From left to right: average AD values of GM, VHs, IZ, DHs, WM, vCST, lCST and FG measured from C3 to C4 in HC (box on the left) and SMA patients (dots on the right). The color of patient dots relates to their position compared to the respective distribution of HC values: blue for patient values within the 25th-75th percentile range, magenta for patient values outside the 25th-75th percentile range but within the HC distribution, red for patient values outside the HC distribution.

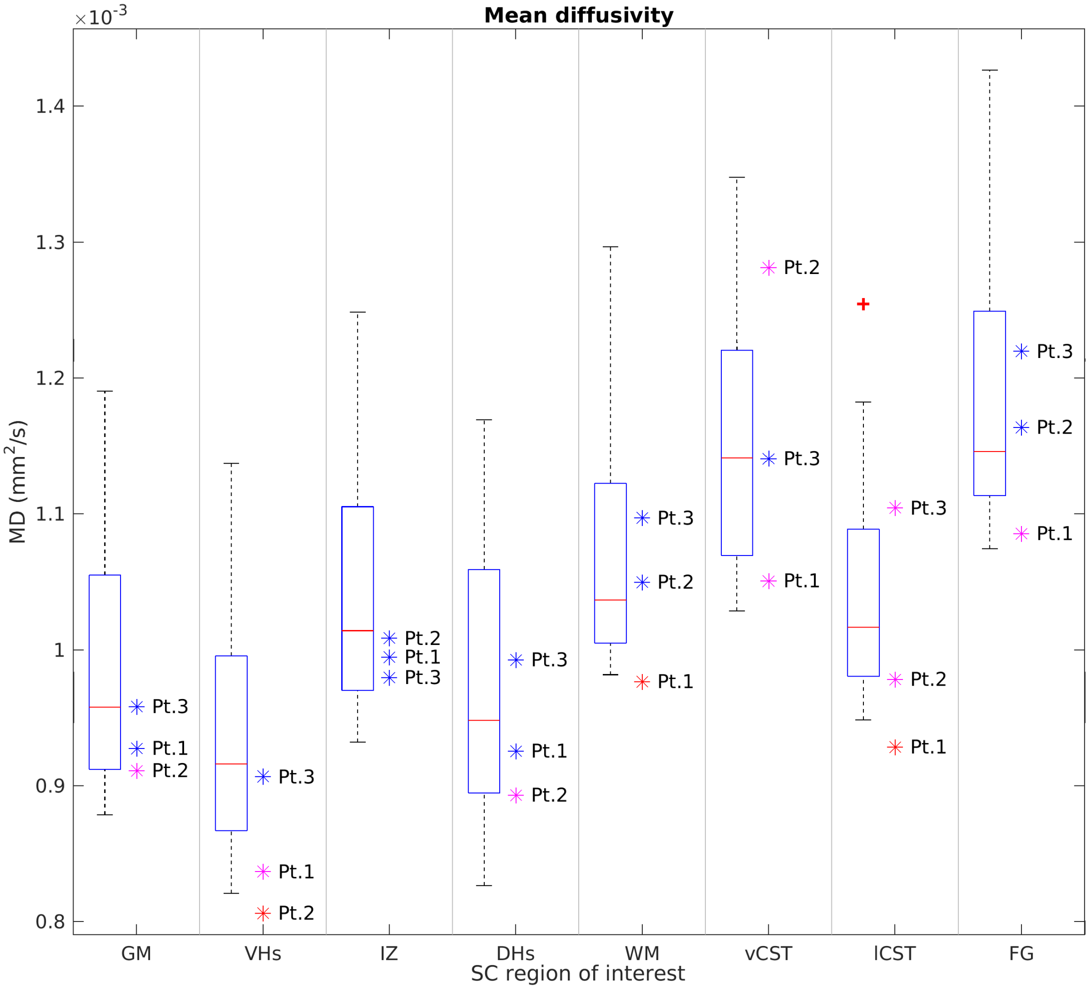

Supplementary Figure 2 - Cross-sectional comparison of average MD values measured at TP3. From left to right: average MD values of GM, VHs, IZ, DHs, WM, vCST, lCST and FG measured from C3 to C4 in HC (box on the left) and SMA patients (dots on the right). The color coding of patient dots is explained in the caption of SupplementaryFigure 1.

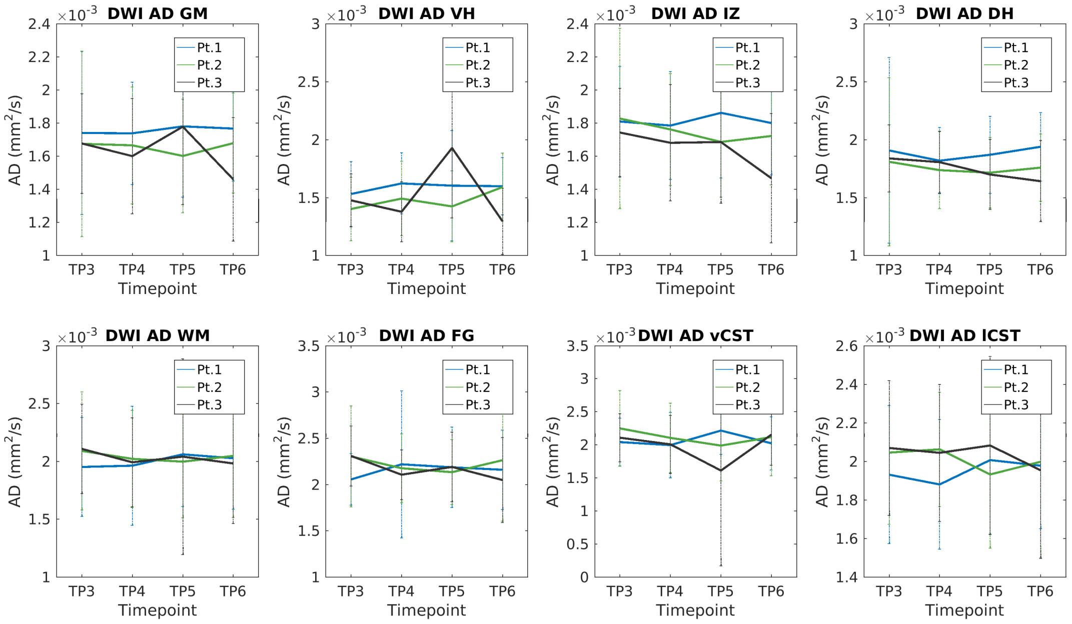

Supplementary Figure 3 - Longitudinal analysis of average AD values. From left to right and top to bottom: average AD values of GM, VHs, IZ, DHs, WM, vCST, lCST and FG measured from C3 to C4 starting from TP3 to TP6.

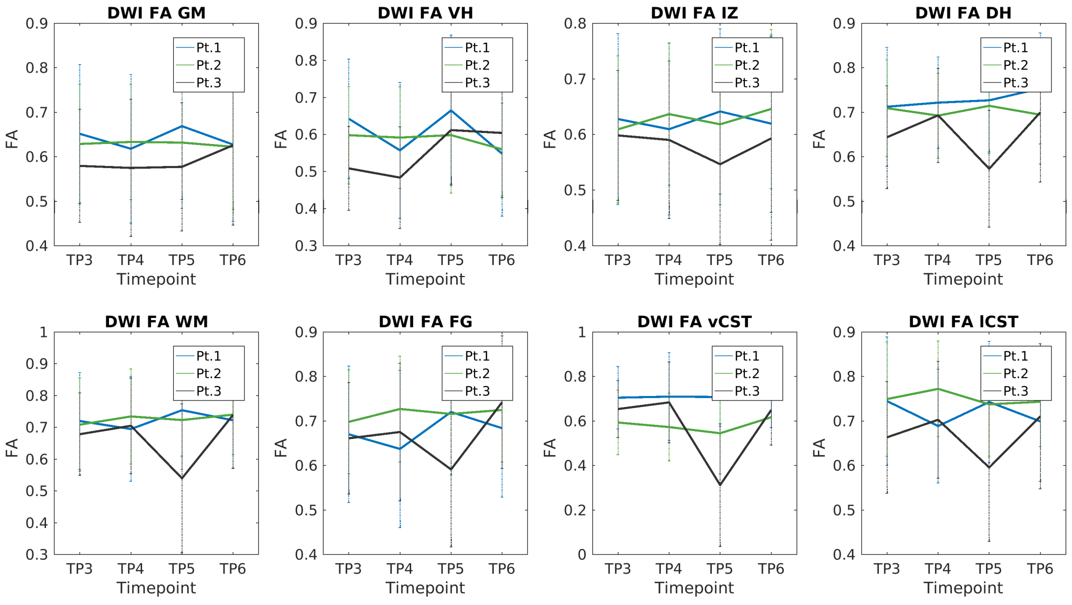

Supplementary Figure 4 - Longitudinal analysis of average FA values. From left to right and top to bottom: average FA values of GM, VHs, IZ, DHs, WM, vCST, lCST and FG measured from C3 to C4 starting from TP3 to TP6.

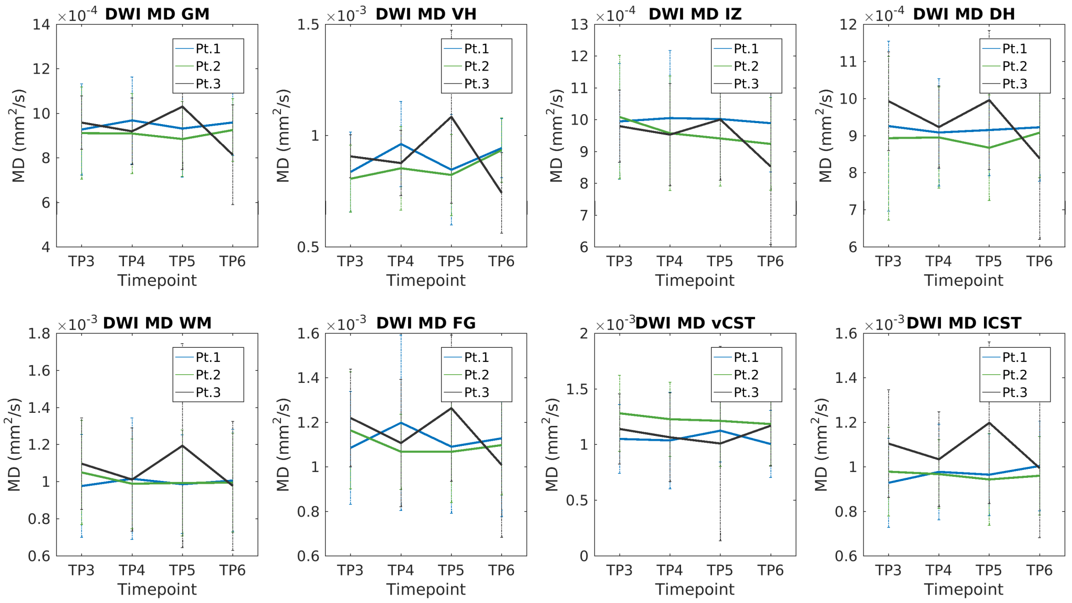

Supplementary Figure 5 - Longitudinal analysis of average MD values. From left to right and top to bottom: average MD values of GM, VHs, IZ, DHs, WM, vCST, lCST and FG measured from C3 to C4 starting from TP3 to TP6.

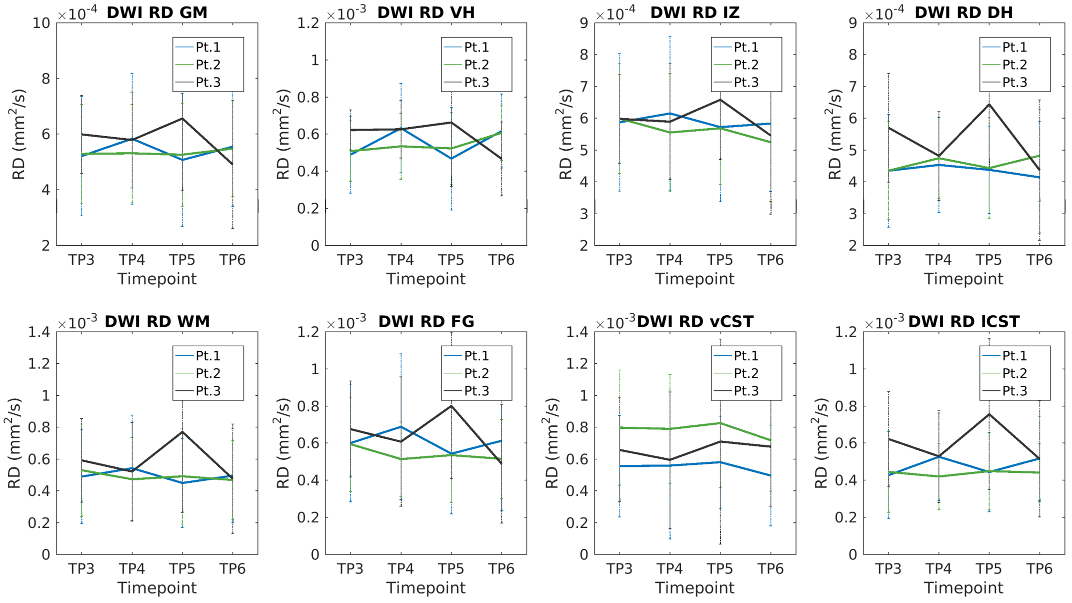

Supplementary Figure 6 - Longitudinal analysis of average RD values From left to right and top to bottom: average RD values of GM, VHs, IZ, DHs, WM, vCST, lCST and FG measured from C3 to C4 starting from TP3 to TP6.
